# Draft Genome Sequence of “*Cohnella kolymensis*” B-2846

**DOI:** 10.1128/genomeA.01587-15

**Published:** 2016-01-14

**Authors:** Andrey V. Karlyshev, Ekaterina B. Kudryashova, Elena V. Ariskina

**Affiliations:** a School of Life Sciences, Pharmacy and Chemistry, Faculty of Science, Engineering and Computing, Kingston University, Kingston upon Thames, United Kingdom; b Skryabin Institute of Biochemistry and Physiology of Microorganisms, Russian Academy of Sciences, Pushchino, Russia

## Abstract

A draft genome sequence of “*Cohnella kolymensis*” strain B-2846 was derived using IonTorrent sequencing technology. The size of the assembly and G+C content were in agreement with those of other species of this genus. Characterization of the genome of a novel species of *Cohnella* will assist in bacterial systematics.

## GENOME ANNOUNCEMENT

Strain 20.16 (VKM B-2846) was isolated from the permafrost using aseptic sampling technique in the borehole drilled in the Duvaniy Yar area, Siberia. This sediment, which was frozen about 5,000 years ago, had been originally formed in a lake during the Holocene optimum period 6,000 to 8,000 years ago. The bacteria were isolated as described previously (1). According to the results of phylogenetic analysis based on 16S rRNA gene sequences, strain B-2846 is most closely related to *Cohnella* spp., with *Cohnella collisoli* showing the highest level of similarity (96.91%). Based on the threshold of 98.65% in recognizing novel species (2), strain B-2846 is regarded as a novel species of *Cohnella* with the proposed name “*Cohnella kolymensis*.”

The sequencing reads were generated by IonTorrent PGM using a 314v2 chip and a 400-bp sequencing kit. Assemblies of reads generated by SPADES plugin ver. 3.1.0 of the Torrent Server (41 contigs) and CLC Genomics Workbench software ver. 7.5 (184 contigs) were combined by using CISA contigs integrator (3) to produce 34 contigs, with the sizes ranging between 1 kb and 765 kb (4,344,717 bp in total, 17.92× genome coverage). The assembly was verified by read mapping.

Both the estimated genome size (4.34 Mb) and G+C content (50.5%) of this strain were slightly lower than these figures for just three other draft genome sequences of other *Cohnella* strains available in the GenBank at the time of preparation of the manuscript (4.5 to 5.0 Mb and 53.5% to 58.3%, respectively). The genome sequence annotation using the RAST server (4) identified 4,731 protein-encoding genes. The number of protein-encoding genes identified by use of the NCBI GenBank genome annotation pipeline was significantly less (3,851). Among the genes found were antibiotic resistance-related genes, including several genes encoding putative beta-lactamases. A gene encoding a bacteriocin biosynthesis protein SagD (645 amino acids [aa]) with up to 65% identity to similar proteins in various *Bacillus* spp. was detected. The bacterium has a propensity to produce a polysaccharide capsule, with a *cpsI* gene product (sugar isomerase/dehydrogenase) also showing only low similarity (up to 57% identity) to proteins found in *Bacillus* spp. Similarly, an EpsL-like exopolysaccharide biosynthesis protein has only 70% sequence identity to the most closely related orthologue in *Cohnella laeviribosi*. Some other gene products shared a bit higher level of similarity to those in various *Cohnella* spp., such as, e.g., a beta-lactamase with 82% identity to that of *Cohnella panacarvi*. Comparative analysis of housekeeping genes *gyrA* and *gyrB* revealed the highest similarity of the respective products to orthologues in *Cohnella thermotolerans* (GyrA, 85% identity) and *Cohnella panacarvi* (GyrB, 87% identity). Note that the identity percentages shown refer to 100% (or close to 100%) subject sequence coverage.

To summarize, the genome sequence of “*Cohnella kolymensis*” B-2846 revealed specific features of this strain, with some genes (16S rRNA, *gyrA*, and *gyrB*) suggesting its close relationship with other *Cohnella* spp., while a number of other genes showed much higher similarity to the genes found in other bacterial genera. The findings are important for studies in the area of bacterial systematics and taxonomy, as well as for phylogenetic analysis.

### Nucleotide sequence accession numbers.

This whole-genome shotgun project has been deposited at DDBJ/EMBL/GenBank under the accession number JXAL00000000. The version described in this paper is version JXAL01000000.
